# Perspectives and practices of dietitians with regards to social/mass media use during the transitions from face-to-face to telenutrition in the time of COVID-19: A cross-sectional survey in 10 Arab countries

**DOI:** 10.3389/fpubh.2023.1151648

**Published:** 2023-05-03

**Authors:** Khlood Bookari, Jamila Arrish, Majid M. Alkhalaf, Mudi H. Alharbi, Sara Zaher, Hawazin M. Alotaibi, Reema Tayyem, Narmeen Al-Awwad, Radwan Qasrawi, Sabika Allehdan, Haleama Al Sabbah, Sana AlMajed, Eiman Al Hinai, Iman Kamel, Jalila El Ati, Ziad Harb, The Arabic Dietitians practices (ADP) survey Group, Maha Hoteit

**Affiliations:** ^1^Department of Clinical Nutrition, Faculty of Applied Medical Sciences, Taibah University, Madinah, Saudi Arabia; ^2^National Nutrition Committee (NNC), Saudi Food and Drug Authority (Saudi FDA), Riyadh, Saudi Arabia; ^3^College of Medicine, King Saud bin Abdulaziz University for Health Sciences, Jeddah, Saudi Arabia; ^4^Department of Human Nutrition, College of Health Sciences, Qatar University, Doha, Qatar; ^5^Department of Clinical Nutrition and Dietetics, Faculty of Applied Health Sciences, Hashemite University, Zarqa, Jordan; ^6^Department of Computer Science, Al-Quds University, Jerusalem, Palestine; ^7^Department of Computer Engineering, Istinye University, Istanbul, Turkey; ^8^Department of Biology, College of Science, University of Bahrain, Sakhir, Bahrain; ^9^Department of Public Health, College of Health Sciences, Abu Dhabi University, Abu Dhabi, United Arab Emirates; ^10^Food and Nutrition Administration, Ministry of Health, Kuwait City, Kuwait; ^11^Dietetics and Nutrition Department, Al Nahdha Hospital, Ministry of Health, Muscat, Oman; ^12^National Research Centre, Cairo, Egypt; ^13^INNTA (National Institute of Nutrition and Food Technology), SURVEN (Nutrition Surveillance and Epidemiology in Tunisia) Research Laboratory, Tunis, Tunisia; ^14^Department of Nutrition, Faculty of Pharmacy, Saint Joseph University, Beirut, Lebanon; ^15^Food Sciences Unit, National Council for Scientific Research (CNRS), Beirut, Lebanon

**Keywords:** COVID-19, dietitians, social media, mass media, telenutrition, barriers, benefits, Arab countries

## Abstract

During the COVID-19 pandemic, most healthcare professionals switched from face-to-face clinical encounters to telehealth. This study sought to investigate the dietitians’ perceptions and practices toward the use of social/mass media platforms amid the transition from face-to-face to telenutrition in the time of COVID-19. This cross-sectional study involving a convenient sample of 2,542 dietitians (mean age = 31.7 ± 9.5; females: 88.2%) was launched in 10 Arab countries between November 2020 and January 2021. Data were collected using an online self-administrated questionnaire. Study findings showed that dietitians’ reliance on telenutrition increased by 11% during the pandemic, *p* = 0.001. Furthermore, 63.0% of them reported adopting telenutrition to cover consultation activities. Instagram was the platform that was most frequently used by 51.7% of dietitians. Dietitians shouldered new difficulties in dispelling nutrition myths during the pandemic (58.2% reported doing so vs. 51.4% pre-pandemic, *p* < 0.001). Compared to the pre-pandemic period, more dietitians perceived the importance of adopting tele nutrition’s clinical and non-clinical services (86.9% vs. 68.0%, *p* = 0.001), with 76.6% being confident in this practice. In addition, 90.0% of the participants received no support from their work facilities for social media usage. Following the COVID-19 outbreak, the majority of dietitians (80.0%) observed a rise in public interest in nutrition-related topics, particularly those pertaining to healthy eating habits (*p* = 0.001), healthy recipes (p = 0.001), nutrition and immunity (p = 0.001), and medical nutrition therapies (*p* = 0.012). Time constraint was the most prevalent barrier to offering telenutrition for nutrition care (32.1%), whereas leveraging a quick and easy information exchange was the most rewarding benefit for 69.3% of the dietitians. In conclusion, to ensure a consistent provision of nutrition care delivery during the COVID-19 pandemic, dietitians working in Arab countries adopted alternative telenutrition approaches through social/mass media.

## 1. Introduction

The COVID-19 pandemic is a global pandemic of coronavirus disease 2019 (COVID-19), caused by severe acute respiratory syndrome coronavirus 2 (SARS-CoV-2) (1). Containment attempts failed, and the virus spread to Arab countries (1). During the COVID-19 period, quarantine measures included closing borders, schools, and public spaces, allowing employees to work remotely (2). Non-emergency surgery and operations were suspended, several face-to-face consultations were postponed or canceled, and healthcare professionals were left uncertain about how to strike a balance between the requirements of society and those of specific patients (3). To guarantee a continuous provision of healthcare services, including consultations, health-related monitoring, and education, many health professionals started adopting telehealth approaches amid confinement which include clinical as well as non-clinical services through using video conferencing, e-mail, mobile or app-enabled technology, and other technologies (4, 5). As for dietitians, “telenutrition,” defined by the American Dietetic Association (ADA), is a subset of telehealth that “involves the interactive use of electronic information and telecommunications technology to implement the nutrition care process (NCP) with remote patients or clients.” (6) In this regard, our earlier survey in five Arab countries found that, during the COVID-19 pandemic, the Arab hospital dietitian’s responsibilities extended beyond the nutrition care setting to include educating the public, answering questions, fostering positive changes in eating habits and teaching them how to do it by disseminating accurate information about nutrition and dispelling myths about it (7). In addition, Arab dietitians developed individualized nutrition plans, offered nutrition support to COVID-19 patients, managed the nutrition-related symptoms of the coronavirus, and prevented the disease’s metabolic complications (7), which are typically linked to obesity, cardiovascular disease, and diabetes (8). However, there is currently a lack of data about the perceptions and practices of dietitians concerning the use of social/mass media amid the transition from face-to-face to telenutrition in the Arab region. Thus, the objective of this study is to examine the perceptions and practices of licensed/registered dietitians regarding social/mass media use in telenutrition in response to the COVID-19 pandemic in 10 Arab countries [Bahrain, Egypt, Jordan, Kuwait, Lebanon, Oman, Palestine, Saudi Arabia, Tunisia, and the United Arab Emirates (UAE)]. In addition, the challenges, opportunities, and benefits encountered with telenutrition were also reported in this study. Many of the lessons from the COVID-19 pandemic in the Arab countries are revealed in our study’s findings, and it is critical to emphasize these lessons when developing future policies for dietitians’ use of telenutrition during endemic and pandemic periods.

## 2. Materials and methods

### 2.1. Study design and recruitment procedure

The present investigation is a cross-sectional study launched in 10 Arab countries: Bahrain, Egypt, Jordan, Kuwait, Lebanon, Oman, Palestine, Saudi Arabia, Tunisia, and the United Arab Emirates (UAE). Between November 2020 and January 2021, when COVID-19 was an emerging pandemic over the world, an online multi-component self-administered questionnaire (34 items) was distributed to dietitians in the aforementioned Arab countries. The survey link was kept open to all licensed/registered dietitians who were residing solely in any of the Arab nations indicated. Participants eligible to participate in this study were those who were licensed or registered according to laws and aged 18 and above.

### 2.2. Questionnaire

A multi-structured questionnaire, designed by experts in the field, was used to meet the study aims. The first part of the questionnaire covered the demographic and professional characteristics of the participants (residence, age, gender, qualifications, and years of expertise in the dietetic practice, highest education level, employment status, and profession). Information in the second part was collected in periods before and during the COVID-19 pandemic, including whether using social media/mass media platforms to provide telenutrition, being active on social media/mass media platforms (with the hours being active and the most frequently used platforms), the reason(s) behind providing telenutrition, whether the dietitians had participated in providing nutrition-related information to the public or other professionals, or attended online nutrition-related courses, and if they responded to nutrition myths on social media. Another section of the questionnaire inquired about how the dietitians perceived the change in the public interest towards nutrition information during the COVID-19 pandemic, and the most nutrition topics being asked about. The last section of the questionnaire explored the barriers and benefits associated with the telenutrition practice.

To ensure its accuracy, the employed questionnaire underwent a face validity (also known as “logic validity”) evaluation. An expert panel made up of dietitians separately assessed each survey question. Additionally, a statistician (APD and expert in question design) analysed the survey for typical mistakes (e.g., leading, confusing, or double-barrelled questions). Each country distributed the employed questionnaire to 14 dietitians among the study’s pilot respondents. Before being made available, the questionnaire underwent pretesting to determine its viability and cultural acceptability. On average, it takes 10–15 min to complete. The questionnaire was completed in Arabic, which was the participants’ mother language.

### 2.3. Ethical considerations

The ethical committee at the University of Taibah, Saudi Arabia has approved the current study protocol. Written informed consent was provided by all participants before filling out the questionnaire. The guidelines outlined in the Declaration of Helsinki were all accounted for while conducting this study. All participants were aware of their rights to voluntarily participate in the study, and that participation refusal or withdrawal was possible at any time, with no need to justify. Each country’s research team was available to answer inquiries about the study. Besides, we collected no identification information of participants.

### 2.4. Statistical analysis

All data were cleaned and exported to the Statistical Package of Social Sciences Software (SPSS; Version 25.0. IBM Corp: Armonk, NY, USA) for analysis. The weighting of cases was performed to improve the representation of samples from each country. Frequencies (*N*) and percentages (%), were obtained for categorical variables, while means and standard deviations (SD) were used to summarize the findings of numerical variables. The chi-squared test (*χ*^2^) was used to determine the associations between study variables. Besides, we used the McNemar test to detect response differences between the two periods, before and during the COVID-19 pandemic. A value of *p* of less than 0.05 was considered significant for all analytical tests.

## 3. Results

### 3.1. Demographic and profession-related characteristics of the study sample

Table 1 shows the demographic and profession-related characteristics of the recruited dietitians. Roughly 88.2% of the total participants were females. Dietitians were recruited from 10 Arab countries: Bahrain, Egypt, Jordan, Kuwait, Lebanon, Oman, Palestine, Saudi Arabia, Tunisia, and UAE. Most of the recruited dietitians were young adults (18–34 years old), 32.6% were middle-aged (32.6%; 35–64 years old), and a few were old adults (>65 years old). Regarding the years of expertise in dietetic practices, almost 19.8% of the participants were newly graduated, nearly half had 2–10 years of experience, and 26.7% had more than 10 years of experience. More than half the participants had Bachelor’s degree, and 25.1% had completed higher studies (Master’s or doctor of philosophy (Ph.D.) degrees). Just 59.0% of the participants had full-time or part-time jobs, 12.3% were self-employed, and 16.4% had no employment. However, the remaining were either retired, working as a volunteer, or still studying. The majority of the dietitians were working in the healthcare sector (54.7%; Table 1).

**Table 1:** Demographic and profession-related characteristics of the study sample.

	Overall (*n* = 2,542)	Female (*n* = 2,243)	Male (*n* = 299)	Value of *p*
	*n* (%)	*n* (%)	*n* (%)	
Country of residence				<0.001*
Bahrain	253 (10.0)	233 (10.4)	20 (6.7)	
Egypt	250 (9.8)	172 (7.7)	78 (26.0)	
Jordan	255 (10.0)	232 (10.4)	23 (7.6)	
Kuwait	256 (10.1)	245 (10.9)	11 (3.8)	
Lebanon	255 (10.0)	250 (11.1)	5 (1.6)	
Oman	255 (10.0)	198 (8.8)	56 (18.8)	
Palestine	254 (10.0)	246 (11.0)	8 (2.6)	
Saudi Arabia	255 (10.0)	224 (10.0)	30 (10.1)	
Tunisia	254 (10.0)	214 (9.5)	40 (13.4)	
UAE	255 (10.0)	227 (10.1)	28 (9.5)	
Age in years				<0.001*
Early adulthood (18–34)	1706 (67.1)	1,539 (71.0)	113 (37.6)	
Middle age (35–64)	827 (32.6)	642 (28.6)	185 (61.9)	
Late adulthood (≥65 years old)	9 (0.3)	7 (0.3)	2 (0.5)	
Years of expertise				<0.001*
Newly graduated	505 (19.8)	483 (21.6)	21 (7.0)	
Less than 2 years	409 (16.1)	385 (17.1)	24 (8.1)	
2–5 years	558 (22.0)	511 (22.8)	48 (15.9)	
6–10 years	391 (15.4)	325 (14.5)	66 (22.2)	
>10 years	679 (26.7)	539 (24.1)	140 (46.8)	
Highest education level				<0.001*
Diploma	352 (13.9)	324 (14.4)	29 (9.5)	
Bachelor	1,551 (61.0)	1,392 (62.1)	158 (52.9)	
Master’s	469 (18.4)	395 (17.6)	74 (24.7)	
Doctor of philosophy (Ph.D.)	170 (6.7)	132 (5.9)	38 (12.8)	
Employment status				<0.001*
Full-time job (40 h./week)	1,195 (47.0)	1,037 (46.2)	158 (53.0)	
Part time job (<40 h./week)	306 (12.0)	278 (12.4)	29 (9.6)	
Unemployed	414 (16.4)	395 (17.6)	19 (6.4)	
Self-employed	313 (12.3)	249 (11.1)	65 (21.6)	
Retired	35 (1.4)	22 (1.0)	13 (4.4)	
Volunteer	61 (2.4)	60 (2.7)	1 (0.3)	
Still studying	218 (8.6)	203 (9.1)	14 (4.8)	
Profession (*n* = 1865)				<0.001*
Healthcare	1,389 (54.7)	1,211 (74.7)	178 (71.4)	
Food/health organizations	191 (7.5)	179 (11.0)	12 (4.9)	
Education (i.e., teaching)	162 (6.4)	143 (8.8)	19 (7.5)	
Food manufacturing	86 (3.4)	57 (3.5)	29 (11.3)	
Agriculture	31 (1.2)	20 (1.2)	11 (4.3)	
Others	6 (0.3)	6 (0.3)	1 (0.3)	

*Significant at value of *p* < 0.05.

### 3.2. Dietitians’ practices and perceptions towards using social media/mass media platforms for telenutrition before and during the COVID-19 pandemic

Table 2 shows that the proportion of dietitians who reported using social media and mass media platforms for telenutrition increased from 68.9% (pre-pandemic period) to 80.0% during the pandemic, *p* < 0.001. This was also manifested by the finding that, compared to the pre-pandemic period, there was around a 20.0% increase in the number of dietitians who considered themselves active on social media in terms of communicating nutrition information during the pandemic, *p* = 0.001. Of interest, amid the COVID-19 outbreak, 4 out of 10 dietitians admitted spending more than 4 h on social media or mass media platforms as a part of their nutrition practice. It is also vivid that the use of different platforms for nutrition communication had increased during the COVID-19 pandemic, of them are Instagram, Twitter, Facebook, Snapchat, LinkedIn, smartphone applications, nutrition blog networks, and many others. Notably, Instagram was the platform that most frequently utilized before and during the pandemic to communicate nutrition information. Providing nutrition consultations, facilitating information sharing and discussion with other professionals, and providing evidence-based nutrition information to the public were the top three ranked reasons by dietitians for telenutrition in both periods before, and during COVID-19 pandemic, value of ps < 0.001. As well, seven out of 10 dietitians participated in providing nutrition-related in-formation to the public or other professionals during the pandemic, which was highest than those reporting so before the pandemic (59.3%), *p* < 0.001. Not only used to provide nutrition communication, but the proportion of dietitians who reported “always” attending online nutrition courses also tripled during the pandemic period. Interestingly, during the pandemic, there was a marked increase in the number of dietitians who reported responding to nutrition myths on social media (58.2%) of the dietitians in contrast to 51.4% prior to the pandemic (Table 2).

**Table 2:** Dietitians ‘practices and perceptions towards using social media/mass media platforms for telehealth communication before and during the COVID-19 pandemic.

	Before COVID-19*n* (%)	During COVID-19*n* (%)	Value of *p*
Using social media/mass media platforms for telehealth communication			<0.001*
No	790 (31.1)	509 (20.0)	
Yes	1752 (68.9)	2033 (80.0)	
Being active on social media/mass media platforms for telehealth communication			<0.001*
No	1,329 (52.3)	814 (32.0)	
Yes	1,213 (47.7)	1728 (68.0)	
Estimated spent hours using social media/mass media platforms for telehealth communication			<0.001*
Less than 1 h	702 (27.6)	339 (13.3)	
1–2 h	698 (27.5)	552 (21.7)	
3–4 h	464 (18.3)	596 (23.4)	
More than 4 h	677 (26.6)	1,055 (41.5)	
The telehealth nutrition communication is a typical part of the job responsibilities			<0.001*
No	1,470 (57.8)	919 (36.2)	
Yes	1,072 (42.2)	1,623 (63.8)	
The most used platform/method for telehealth communication			
Instagram	1,198 (47.1)	1,313 (51.7)	<0.001*
Twitter	470 (18.5)	537 (21.1)	<0.001*
Facebook	960 (37.8)	1,041 (40.9)	<0.001*
Snapchat	387 (15.2)	478 (18.8)	<0.001*
Mobile calls	574 (22.6)	715 (28.1)	<0.001*
WhatsApp	70 (2.7)	57 (2.2)	<0.001*
Emails	523 (20.6)	616 (24.2)	<0.001*
Smart phone applications (i.e., Zoom, Microsoft teams, Skype, …)	473 (18.6)	778 (30.6)	<0.001*
YouTube	308 (12.1)	430 (16.9)	<0.001*
Telegram	187 (7.3)	248 (9.8)	<0.001*
LinkedIn	153 (6.0)	210 (8.3)	<0.001*
Nutrition blog networks	127 (5.0)	169 (6.7)	<0.001*
Electronic newspaper/magazines	208 (8.2)	260 (10.2)	<0.001*
Pinterest	94 (3.7)	141 (5.5)	<0.001*
Personal blogs	104 (4.1)	136 (5.3)	<0.001*
Television programs	235 (9.2)	277 (10.9)	<0.001*
Radio channels	162 (6.4)	199 (7.8)	<0.001*
Interactive websites	88 (3.4)	141 (5.5)	<0.001*
Discussion forums	83 (3.3)	143 (5.6)	<0.001*
Collaborative projects (i.e., Wikis)	65 (2.5)	93 (3.7)	<0.001*
Virtual social world (e.g., Linden Lab, San Francisco, California, …)	38 (1.5)	75 (2.9)	<0.001*
The reason behind providing remote nutrition communication			
Provide nutrition consultation	1,300 (51.1)	1,602 (63.0)	<0.001*
Facilitate information sharing/discussion with other professionals	841 (33.1)	1,177 (46.3)	<0.001*
Provide evidence-based nutrition information to the public	908 (35.7)	1,122 (44.1)	<0.001*
Spread organization news	378 (14.9)	438 (17.2)	<0.001*
Advertise private business (i.e., clinic)	339 (13.3)	426 (16.8)	<0.001*
Publish education materials or conduct training courses	338 (13.3)	552 (21.7)	<0.001*
Part of onsite coaching and training workshops	319 (12.5)	537 (21.1)	<0.001*
Create video content for public (nutrition-related)	412 (16.2)	595 (23.4)	<0.001*
Showcase food and recipes	446 (17.5)	549 (21.6)	<0.001*
Participating in providing nutrition-related information to the public or other professionals			<0.001*
No	1,035 (40.7)	760 (29.9)	
Yes	1,508 (59.3)	1782 (70.1)	
Attending online nutrition-related courses			<0.001*
Never	600 (23.6)	279 (11.0)	
Rarely	873 (34.3)	369 (14.5)	
Often	787 (31.0)	1,009 (39.7)	
Always	282 (11.1)	885 (34.8)	
Responding to nutrition myths on the social media			<0.001*
No	1,235 (48.6)	1,064 (41.8)	
Yes	1,307 (51.4)	1,479 (58.2)	
Perceiving the importance of using social media/mass media platforms for telehealth communication			<0.001*
No	817 (32.0)	334 (13.1)	
Yes	1725 (68.0)	2,208 (86.9)	
Being confident (satisfied) in using social media/mass media platforms for telehealth communication			<0.001*
No	991 (39.0)	594 (23.4)	
Yes	1,551 (61.0)	1948 (76.6)	

* Significant at value of *p* < 0.05 for McNemar test.

**Figure 1:**
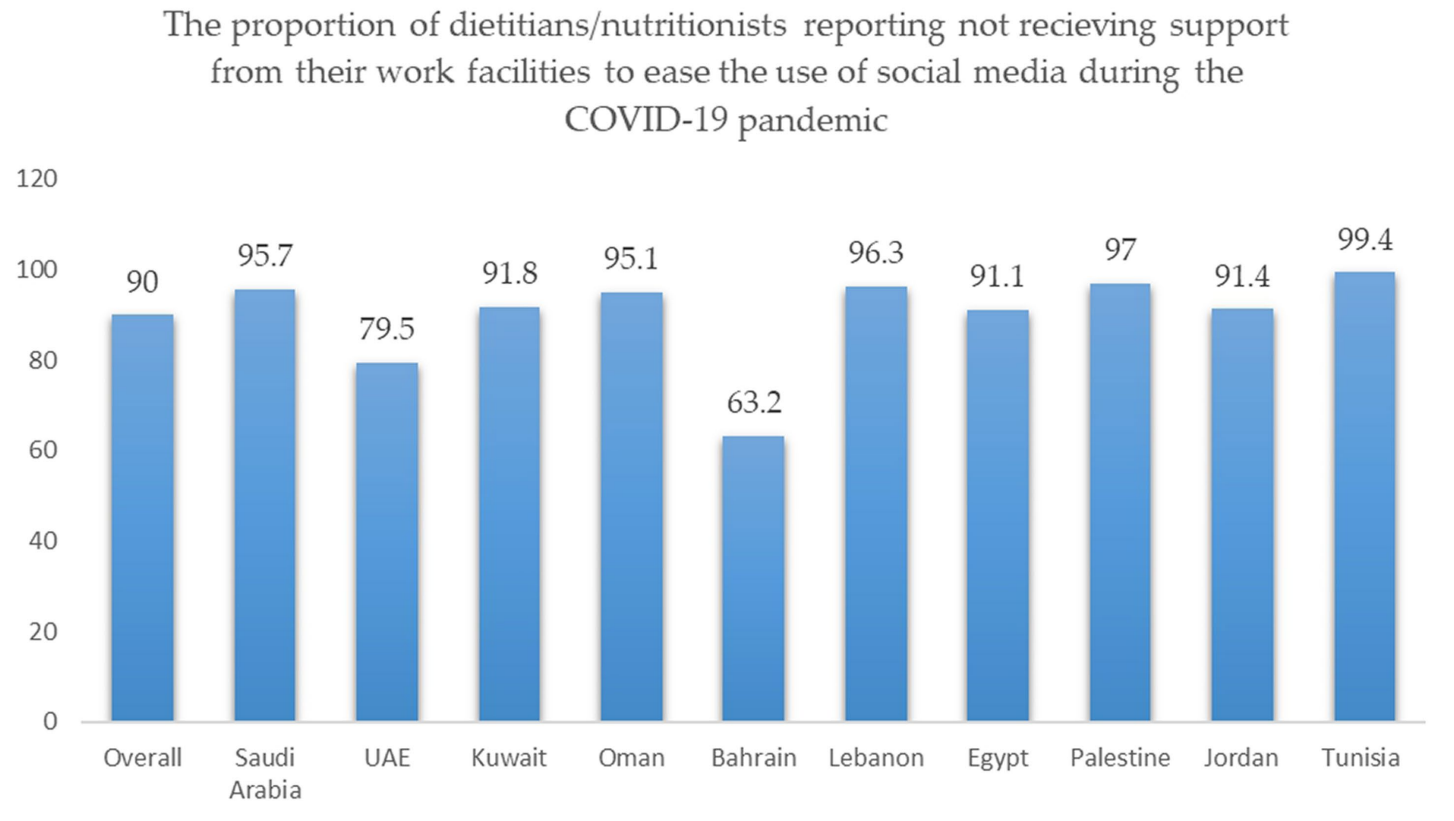
The proportion of dietitians reporting not receiving support from their work facilities to ease the use of social media during the COVID-19 pandemic.

When asked about their perceptions of the importance of using social media/mass media for telenutrition, most of the dietitians rated it important during the pandemic compared to 68.0% (before the pandemic), *p* < 0.001. Furthermore, during the pandemic, 76.6% were confident in its use, exceeding the proportion of those who were confident before the pandemic (61.0%), *p* < 0.001. Nonetheless, as shown in the figure below (Figure 1), almost all the dietitians claimed that they did not receive support from their work facilities to ease the usage of social media during the COVID-19 pandemic (i.e., subscriptions in paid applications), particularly in Tunisia, Palestine, Lebanon, and Saudi Arabia (Table 2).

### 3.3. Dietitians ‘perspectives on the public’s interest in nutrition-related information during the COVID-19 pandemic

**Figure 2:**
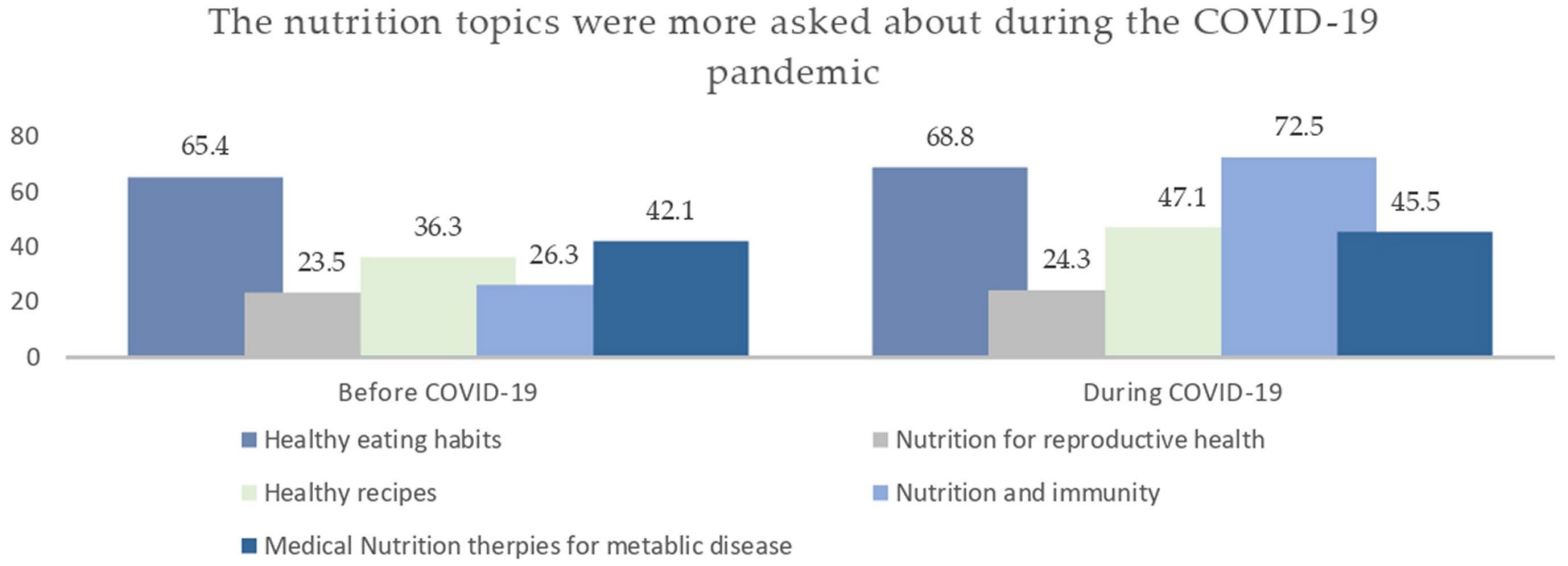
The hot nutrition topics that were more asked during the COVID-19 pandemic.

The participants were asked about their perspectives on the public’s interest in nutrition-related information during the COVID-19 pandemic, and the most frequent nutrition topics were asked about, before and during the pandemic. Around 80.0% of the dietitians perceived an increase in the public’s interest in nutrition-related information since the start of the COVID-19 outbreak (Data not shown). Figure 2 displays the nutritional topics that were asked more frequently during the pandemic than they were before it. Of relevance, there was an increase in public’s interests towards nutrition topics related to healthy eating habits, nutrition for reproductive health, healthy recipes, nutrition and immunity, and medical nutrition therapies (MNTs; pre-pandemic: 42.1%, during the pandemic: 45.5%, *p* = 0.012). (Figure 2).

### 3.4. Barriers and benefits of using social media and mass media platforms to provide telenutrition communication

**Figure 3:**
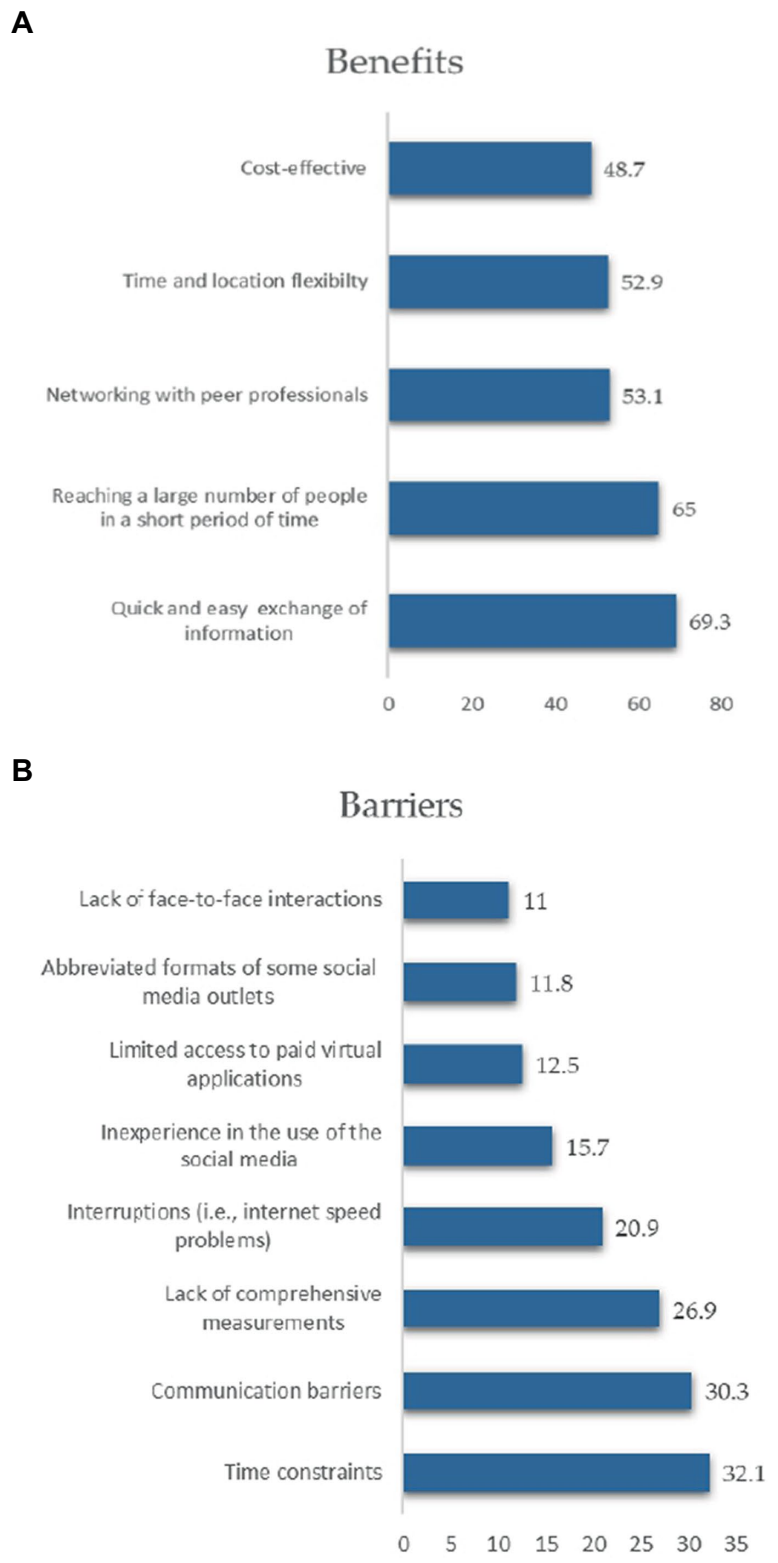
**(A)** The benefits of using social media/mass media platforms to provide telenutrition. **(B)** The barriers of using social media/mass media platforms to provide telenutrition.

Participants were asked to report the most frequent barriers and benefits of using social media/mass media platforms to communicate nutrition information remotely. The highest proportion of dietitians (32.1%) found that time constraints were the most serious obstacle to using social media and mass media in their nutrition and dietetic practice. Other barriers were as follows: communication barriers (30.3%), lack of comprehensive measurements (i.e., anthropometry; 26.9%), interruptions (i.e., connectivity issues; 20.9%), inexperience in the use of social media (15.7%), limited access to paid virtual applications (12.5%), abbreviated formats of some social media outlets (11.8%), and lack of face-to-face interactions (11.0%; Figure 3A) However, the use of social media and mass media was not without benefits for dietitians. Allowing a quick and easy exchange of information was the most perceived benefit by 69.3% of participants, followed by the ability to reach a large number of people in a short period (65.0%), networking with peer professionals (53.1%), time and location flexibility (52.9%), and being cost-effective (48.7%; Figure 3B)

## 4. Discussion

This study, the first of its kind in the Arab region, assessed the dietitians’ perceptions and practices toward the use of social media and mass media platforms for telenutrition during the COVID-19 pandemic. In addition, the encountered barriers and benefits were also reported in this study. Our survey showed an increase of 11% in the percentage of dietitians who reported using social media and mass media platforms to provide telenutrition during the COVID-19 pandemic. This is in line with a preliminary finding that showed that after the pandemic, more than half of dietitians transitioned to providing nutrition consultations over the phone (72.0%) or online databases (54.0%) (2). Conjointly, it supports what has been observed in the United States (9) and Italy (10), where RD respondents reported a rise in the use of telenutrition patient care during the pandemic. RDs even anticipated using telenutrition to establish nutrition consults in post-pandemic care (9). One study also found evidence in the use of telenutrition strategies by RDs for the MNTs of patients with inherited metabolic disorders during the COVID-19 pandemic (11). Dietitians were compelled to find high-quality and efficient alternatives for hospital or clinic consultations owing to the challenges posed by the COVID-19 health crisis (11). Thus, distant consultations were a possibility. During telenutrition visits, RDNs have the option of spending more time with their patients and learning more about their eating habits by gaining access to kitchen cabinets or refrigerators (9). Telenutrition interventions had also been shown to improve weight loss outcomes among cardiovascular disease patients and other patients with obesity (12). Evidence suggests that nutrition outcomes obtained through telenutrition consultations may be on par with those obtained through in-person meetings with patients (13). Dietetic consultations given *via* telenutrition yield results that are comparable to those of in-person consultations without necessitating a higher level of specialized training or lowering the standard of service delivery (13). A responsive and cost-effective replacement or addition to the conventional in-person delivery of dietetic services (13). Successful telenutrition implementation can allow persons with chronic diseases to optimize their diet-related health and well-being, regardless of their location, income, or literacy level, thereby eliminating present injustices (13).

It is also evident in the present study that the use of social media networks (Instagram, Facebook, Twitter, Snap-chat, and others) to provide nutrition communication with patients increased significantly during the pandemic period, and they were mostly used compared to other approaches. Besides, considerable proportions of dietitians also reported a significant increase in the reliance on mobile calls, email messages, and smartphone applications (Zoom, Microsoft Teams, for instance) to pursue their dietetic practice remotely. Notably, Instagram was the platform that dietitians and nutritionists most frequently utilized, as also shown in this study. This is in alignment with data reported among dietitians in Brazil, where almost all of them (91.7%) reported using social networks for nutrition practice, and Instagram was the social network most often used by 85.0% of the dietitians (14). Social media is a platform for creating and maintaining connections (14). This offers a chance to draw patients and clients who are new to telenutrition consultations of any kind (14). During the COVID-19 outbreak, many clinicians including dietitians have used accessible social media sites like TikTok, Instagram, Facebook, and telemedicine platforms and websites, depending on the age and preferences of potential or existing clients (14). However, social media and mass media use must abide by privacy and security laws to be safe enough for clinical encounters (15). For instance, in the United States, video conferencing must com-ply with the Health Insurance Portability and Accountability Act of 1996 for the consultation to be covered by Medicare or Medicaid benefits (15). Unfortunately, most Arab countries did not consider or implement such privacy and security regulations; therefore, attempting to adapt them in the future could undoubtedly promote higher-quality and safer consultations for both dietitians and patients.

When asked about the reasons behind the use of social media and mass media during the COVID-19 pandemic, the majority of our participants reported that it was to provide nutrition consultations, facilitate information sharing and discussion with other professionals, and provide evidence-based nutrition information to the public. This is further demonstrated by the findings that among the benefits reported by dietitians to relying on telenutrition during the pandemic was allowing a quick and easy exchange of information (69.3% of participants), the ability to reach a large number of people in a short period (65.0%), networking with peer professionals (53.1%), time and location flexibility (52.9%), and being cost-effective (48.7%). One study found that RDs were satisfied with using telenutrition consults methods as it allows them to picture the patient’s home environment (3). This finding aligned with our observations, as this could help understand the patient’s eating habits with their promoters in a quick and accessible way (16). Group telenutrition consults have been shown to efficiently deliver nutrition care, including, for example, the integration of multiple family members in a single consultation (16), supporting our finding in terms of allowing to reach a large number of people in a short period; “reaching more people with technology.” This is especially important because previous research has shown that family support help to improve patient comprehension and acceptance of nutrition interventions (16). Telenutrition provided benefits to patients as well, including an increased likelihood of following up with the dietitian and other logistical benefits such as erasing the need to travel, pay for parking, or wait for a long time in medical and clinical settings (10). Contrarily, this study identifies some obstacles to offering telehealth communication through social media and mass media, including time constraints, communication barriers, lack of comprehensive measurements, interruptions (connectivity issues), inexperience in the use of social media, limited access to paid virtual applications, abbreviated formats of some social media outlets, and lack of face-to-face interactions.

Also noteworthy, almost all our participants claimed that their work facilities had not supported the usage of social media (i.e., subscriptions in paid applications) during the COVID-19 pandemic, which might contribute to shaping some of the reported obstacles impeding the effective and efficient use of telehealth consultation approaches among dietitians. Our findings corroborated data in the literature which documents challenges and obstacles while adopting telehealth approaches in providing nutrition care during the COVID-19 pandemic. One recent study showed that dietitians were challenged with the lack needed technology access and connectivity constraints (17). Besides, lack of client interest (29%) and Internet access (26%) as well as the difficulty to conduct or evaluate standard nutrition assessment or monitoring/evaluation activities (28%), were the most often cited hurdles to providing nutrition treatment *via* telenutrition among American nutritionists (9). On the other hand, promoting conformity with social distance (66%) and scheduling flexibility (50%) were two often noted advantages among them (18). In closing, institutions must provide a welcoming and favorable environment for dietitians to practice their profession remotely, with the greatest possible benefits and the fewest possible withdrawals. Moreover, dietitians shouldered new responsibilities in responding to nutrition myths during the pandemic period (7). This is demonstrated by the results of the current study, which found that during the pandemic, there was a marked increase in the number of dietitians who reported responding to nutrition myths on social media. In this context, one study revealed that “immune boosting” was a trending topic during the COVID-19 pandemic, described as “selling immunity on Instagram” (19). What is distressing about this is that “immune boosting” is a scientifically misleading term that was frequently used during the pandemic to market unproven products and treatments, especially dietary supplements (19). Our study supports the literature showing that, during the pandemic, there was a roughly 46.0% increase in the number of people who expressed interest in nutrition and immunity-related topics (19). Google Trends analysis revealed that in February 2020, searches for the terms “immune boost” and “immune boosting” significantly increased (20). Additionally, Instagram posts using the trending hashtag “immune booster” increased by over 46% between April and May 2020 (20). Thereby, dietitian’s responsibilities in protecting the public health from such nutrition-related myths had propelled them into additional stress and challenges.

### 4.1. Study limitations and strengths

The authors acknowledge that this study has some limitations. Firstly, the cross-sectional design does not allow reaching causal inferences. The online nature of the questionnaire, as well, could lead to reporting bias, as some questions might not be well understood; however, given that the study was launched during the lockdown period of COVID-19, this was inevitable. Besides, due to the fact that our participants are dietitians and nutritionists, information bias is minimally expected as they all had university education level. Another limitation is the low participation of male and elderly respondents, as most of our participants are female adults. On the other hand, as far as we know, this is the first study of its kind in the Arab region. Our study’s large sample size also enabled more confident generalizability of its results.

## 5. Conclusion

To ensure a consistent provision of nutrition care delivery during the COVID-19 pandemic, dietitians working in Arab countries adopted alternative telenutrition approaches *via* social media and mass media platforms. Although their telenutrition practices was beneficial in certain circumstances, such as fostering quick and easy discussions and communication, challenges were also prevalent, including those related to time constraints. This study showed that dietitians’ reliance on telenutrition increased during the pandemic. In addition, dietitians shouldered new difficulties in dispelling nutrition myths after the emergence of COVID-19 pandemic. Besides, following the COVID-19 outbreak, the majority of dietitians observed a rise in public interest in nutrition-related topics, particularly those pertaining to healthy eating habits, healthy recipes, nutrition and immunity, and medical nutrition therapies. Nonetheless, many received no support from their working facilities to ease and promote consistent, effective, and efficient telehealth practice. To wrap up, Despite the pandemic’s effect on people’s economic and health status, it may have allowed health care providers like dietitians to reach more people, spreading awareness among them and providing nutritional health care on a wider scale than what it was in the past, confined to the personal presence of the facility health. Work facilities and institutions in the Arab countries should take advantage of this opportunity and provide a welcoming and favorable environment for dietitians to practice their profession remotely, with the greatest possible benefits and the fewest possible withdrawals. Due to the numerous benefits they offer when used properly, telenutrition should not be only associated with pandemic periods.

## The Arabic dietitians practices (ADP) survey group

Jordan: Linda Abu Hawileh (Linda Nutrition Center); Palestine: Malak Amro and Diala Abu Al-Halawa (Al-Quds University); Bahrain: Buthaina Ajlan (Nutrition Section in Public Health Directorate, Ministry of Health, Bahrain); Fatima Alhaddad (Salmaniya Medical Complex, Manama, Bahrain); United Arab Emirates: Leila Cheikh Ismail (Sharjah University); Ayesha S. Aldhaheri (United Arab Emirates University); Kuwait: Maryam AlDwairji, Nawal M. Alqoud (Ministry of Health, Kuwait City 12,009, Kuwait); Oman: Nahla Al Anqodi (Sultan Qaboos University Department of food Science and Nu-trition); Tunisia: Houda Ben Gharbia and Sonia Sassi [INNTA (National Institute of Nutrition and Food Technology), SURVEN (Nutrition Surveillance and Epide-miology in Tunisia) Research Laboratory, Tunis 1,007, Tunisia]; Lebanon: Fatima Al Batoul Hussein and Vanessa Abi Akl (Faculty of Public Health, Lebanese university).

## Data availability statement

The raw data supporting the conclusions of this article will be made available by the authors, without undue reservation.

## Ethics statement

The ethical committee at the University of Taibah, Saudi Arabia has approved the current study protocol. Written informed consent was provided by all participants before filling out the questionnaire.

## Author contributions

KB, JA, MaA, MuA, SZ, and H-wA: conceptualization. KB, JA, MaA, MuA, SZ, and HA: methodology. KB, JA, and HA: formal analysis. KB, JA, MaA, MuA, SZ, HA, RT, NA-A, RQ, SbA, HS, EA, IK, JE, ZH, and MH: investiga-tion. KB, JA, and MH: data curation. KB: writing—original draft preparation, visualization, and supervision. KB, RT, RQ, SbA, HS, EA, IK, JE, and MH: writing—review and editing. SnA: helped with investigation, writing — review and editing, final approval of the version. All authors contributed to the article and approved the submitted version.

## Conflict of interest

The authors declare that the research was conducted in the absence of any commercial or financial relationships that could be construed as a potential conflict of interest. The author disclaim that the views expressed in this article do not necessarily represents the views, decisions or policies of Saudi FDA or other institutions with which the authors are affiliated.

## Publisher’s note

All claims expressed in this article are solely those of the authors and do not necessarily represent those of their affiliated organizations, or those of the publisher, the editors and the reviewers. Any product that may be evaluated in this article, or claim that may be made by its manufacturer, is not guaranteed or endorsed by the publisher.
