# Supplementary material for: Perspectives and practices of dietitians with regards to social/mass media use during the transitions from face-to-face to telenutrition in the time of COVID-19: A cross-sectional survey in 10 Arab countries
Source: Front Public Health. 2023 May 3;11:1151648. doi: 10.3389/fpubh.2023.1151648 (PMC10208426; doi:10.3389/fpubh.2023.1151648)
Supplement: Supplementary file 1 [file Table_1.DOCX]

**Table S1.** Study Instrument: Questionnaire

| **1. Giving consent**   - I have read the consent form and I agree to participate in this survey - I do not agree to participate in this survey |  |
| --- | --- |
| **2. Which Arabic country are you residing/working in?**   - Lebanon - Egypt - Palestine - Jordan - Tunisia - Saudi Arabia - Kuwait - United Arab Emirates - Oman - Bahrain | **3. Please select your gender**   - Female - Male |
| **4. What is your age?............** | **5. How many years of expertise do you have in nutrition/dietetics practice?**   - Newly graduated - Less than two years - 2-5 years - 6-10 - More than 10 years |
| **6. What is your highest degree of education?**   - Diploma - Bachelor’s - Master’s - PhD | **7. What is your current employment status?**   - Full time (40+ hr./w) - Part time (< 40 hr./w) - Unemployed (looking for work) - Unemployed (not looking for work) - Student - Retired - Self-employed - I work as a volunteer - Unable to work |
| **8. What nutritional related ﬁeld do you work in?**   - Education (i.e. teaching) - Food Manufacturing - Healthcare - Agriculture - Food and/health organizations   Other (please specify)…………. |  |
| 1. ***Definition of Nutrition-Related Practice:***   Nutrition-Related Practice includes using professional knowledge in both clinical and non-clinical relationships with patients or clients, communities and populations and can be working in management, administration, education, research, advisory, communication, program development and implementation, regulatory or policy development, food service, food security, food supply, sustainability and any other roles that impact on safe, effective delivery of services in the profession and/or using professional skills.  Please note that: practices of dietitians and nutritionists may differ, each should know their expected roles and practices.   1. ***Social media***   Is a broad example of Web and refers to Internet-based platforms devoted to blogging, social networking, collaborative writing projects, content communities, and virtual social worlds   1. ***Active social media***   In relation to dentitions/nutritionists, they share successful experiences of clients; create text, audio, or video content all related to food and nutrition; and respond frequently to other (peer professionals, clients, audience)   1. ***Definition of Telenutrtion****:*   Is when a registered dietitian (RD) provides medical nutrition therapy to patients via online platforms such as Zoom, Google Meets, or Skype. | |
| **9. Before the spread of COVID-19 pandemic, did you use social media/mass media/platforms for telenutrition in your nutrition-related practice?**   - Yes - No | **10. After the spread of COVID-19 pandemic, do you use social media /mass media/platforms for telenutrition in your nutrition-related practice?**   - Yes - No |
| **11. Before the spread of COVID-19 pandemic, did you consider yourself active in using social media /mass media/ platforms for telenutrition in your nutrition-related practice?**   - Yes - No | **12. After the spread of COVID-19 pandemic, do you consider yourself active in using social media /mass media/ platforms for telenutrition in your nutrition-related practice?**   - Yes - No |
| **13. Before the spread of COVID-19 pandemic, roughly how many hours per day did you spend using social media/mass media/platforms for telenutrition in your nutrition-related practice?**   - Less than one hour - 1-2 hours per day - 3-4 hours per day - 5-6 hours per day - 7-8 hours per day - More than 8 hours per day - I did not spend any time in using social media in my nutrition-related practice | **14. After the spread of COVID-19 pandemic, roughly how many hours per day do you spend using social media/mass media/platforms for telenutrition in your nutrition-related practice?**   - Less than one hour - 1-2 hours per day - 3-4 hours per day - 5-6 hours per day - 7-8 hours per day - More than 8 hours per day - I do not spend any time in using social media in my nutrition-related practice |
| **15. Before the spread of COVID-19 pandemic, was using social media/mass media/platforms for telenutrition in your nutrition-related practice a part of your job responsibilities?**   - Yes - No | **16. After the spread of COVID-19, does providing nutrition consultation/advice to individuals or groups via telephone or audio-video (Zoom, FaceTime) become part of your job responsibilities?**   - Yes, it was part of my job responsibility - No, it was voluntarily - I did not use it before |
| **17. Before the spread of COVID-19, If you used social media/mass media/platforms for telenutrition in your nutrition-related practice, what kind of platform did you mostly rely on? Select all that apply.**   - Twitter - Facebook - Instagram - Pinterest - Snapchat - Telegram - Personal blogs - Nutrition Blog Network - Collaborative projects (e. g. wikis) - Virtual social worlds (e. g. Second Life; Linden Lab, San Francisco, California) - Discussion forums - Interactive websites - LinkedIn - YouTube - Tv - Radio - Electronic newspapers/magazines - Telephone - Smart phones applications (e.g. zoom, skype, Microsoft team…) - Emails - I did not use social media/mass media/platforms for telenutrition in my nutrition-related practice - Other (please specify)……… | **18. After the spread of COVID-19, If you use social media/mass media/platforms for telenutrition in your nutrition-related practice, what kind of platform do you mostly rely on? Select all that apply.**   - Twitter - Facebook - Instagram - Pinterest - Snapchat - Telegram - Personal blogs - Nutrition Blog Network - Collaborative projects (e. g. wikis) - Virtual social worlds (e. g. Second Life; Linden Lab, San Francisco, California) - Discussion forums - interactive websites - LinkedIn - YouTube - Tv - Radio - Electronic newspapers/magazines - Telephone - Smart phones applications (e.g. zoom, skype, Microsoft team…) - Emails - I do not use social media/mass media/platforms for telenutrition in my nutrition-related practice - Other (please specify)…… |
| **19. Before the spread of COVID-19 pandemic, if you used media/mass media/platforms for telenutrition in your nutrition-related practice, what purpose did you use it for? Select all that apply.**   - To provide nutrition consultation/advice to individuals or groups - To facilitate sharing and discussion of food and nutrition information/recommendations with other professionals - To facilitate accurate and evidence based nutrition information provision to the general public - To spread organization news - To advertise private business (i.e. personal/virtual clinic; promoting personal brands to the public.) - To conduct and publish an educational materials or training courses for continuing education opportunities - To be used as part of onsite coaching and training workshops. - To create video content available for public (relating to nutrition) - To showcase food and recipes - I did not use social media in my nutrition-related practice - Other (please specify)…….. | **20. After the spread of COVID-19 pandemic, if you use media/mass media/platforms for telenutrition in your nutrition-related practice, what purpose do you use it for? Select all that apply.**   - To provide nutrition consultation/advice to individuals or groups - To facilitate sharing and discussion of food and nutrition information/recommendations with other professionals - To facilitate accurate and evidence based nutrition information provision to the general public - To spread organization news - To advertise private business (i.e. personal/virtual clinic; promoting personal brands to the public.) - To conduct and publish an educational materials or training courses for continuing education opportunities - To be used as part of onsite coaching and training workshops. - To create video content available for public (relating to nutrition) - To showcase food and recipes - I do not use social media in my nutrition related practice - Other (please specify) |
| **21. Do you think the public interest in nutrition information has increased after the spread of COVID-19?**   - Yes - No - I don’t know |  |
| **22. Before the spread of COVID-19 pandemic, what was/were the most frequently nutritional topic/s you have been asked about? Select all that apply.**   - Healthy eating - Nutrition for reproductive health (preconception and fertility, pregnancy, postpartum, lactation) - Nutrition in the life cycle (infancy and childhood; adolescence; adulthood; elderly) - Nutrition for health and ﬁtness - Healthy recipe ideas - Nutrition and immunity - Fad diet (e.g. Paleo diet, The zone diet, Ketogenic diet, Dukan diet, Cabbage soup diet) - Vegetarian and vegan diet - Weight management (i.e. weight loss, weight gain and weight maintenance) - Diabetes management - Nutritional management of cardiovascular disease - Nutritional management of Celiac disease - Food allergy and intolerance - Other medical nutrition therapy   Other (please specify)……… | **23. After the COVID-19 pandemic, what is/are the most frequently nutritional topic/s you are asked about? Select all that apply.**   - Healthy eating - Nutrition for reproductive health (preconception and fertility, conception, pregnancy, postpartum, lactation) - Nutrition in the life cycle (infancy and childhood; adolescence; adult; elderly) - Nutrition for health and ﬁtness - Healthy recipe ideas - Nutrition and immunity - Fad diet (e.g. Paleo diet, The zone diet, Ketogenic diet, Dukan diet, Cabbage soup diet) - Vegetarian and vegan diet - Weight management (i.e. weight loss, weight gain and weight maintenance) - Diabetes management - Nutritional management of cardiovascular disease - Nutritional management of Celiac disease - Food allergy and intolerance - Other medical nutrition therapy - Other (please specify) ……. |
| **24. Before the spread of COVID-19 pandemic, how conﬁdent were you in using social media/mass media/platforms for telenutrition as an integrated part of your nutrition-related practice?**   - Not conﬁdent at all - Slightly conﬁdent - Conﬁdent - Very conﬁdent N/A | **25. After the COVID-19 pandemic, how conﬁdent are you in using social media/mass media/platforms for telenutrition as an integrated part of your nutrition-related practice?**   - Not conﬁdent at all - Slightly conﬁdent - Conﬁdent - Very conﬁdent N/A |
| **26. After the spread of COVID-19, how often do you take online nutrition related courses (online lectures, webinars)?**   - Never - Rarely - Often - Always |  |
| **27. Before the spread of COVID-19, did you participate in providing evidence based nutrition related information to the public and/or dietitians/nutritionists/other health care providers via social media platforms or lectures in your facility or online?**   - Yes - No | **28. After the spread of COVID-19, have you participated in providing evidence based nutrition related information about COVID-19 to the public and/or dietitians/nutritionists/other health care providers via social media platforms or lectures in your facility or online?**   - Yes - No |
| **29. Before the spread of COVID-19, did you respond to false nutrition information on the social media?**   - Yes, I corrected false information, or nutrition myths - No, because I didn't respond to false information, or nutrition myths - No, because I didn’t have the opportunity to respond - No, because I didn’t come across any false nutrition related information - No, because I didn't have enough conﬁdence to respond - I was not interested to respond | **30. After the spread of COVID-19, do you respond to false nutrition information and myths about COVID-19 on the social media?**   - Yes, I correct false nutrition information and myths about COVID-19 if I come across them - No, I don't respond to false nutrition information and myths about COVID-19 if I come across them - No, I don’t have opportunity to respond - No, I don’t come across any false nutrition information and myths about COVID-19 - No, I don't have enough conﬁdence to respond to false nutrition information and myths about COVID-19 - No, I don't have enough information to respond to false nutrition information and myths about COVID-19 - I am not interested to respond |
| **31. Since the beginning of COVID-19 lockdown measures, has your work facilitated the usage of social media. (i.e. subscriptions in paid applications etc.)**   - No - Yes (give examples) | **32. In your opinion what is/are the barriers you experience in using social media in your nutrition-related practice? Select all that apply.**   - Communication barriers (missing out on a lot of non-verbal communication cues) - Abbreviated formats of some social media outlets, such as Twitter's 140character limit - Time constrains - Inexperience in the usage of social media - Interruptions in internet access - Inexperience in virtual class usage due to lack of face-to-face interactions - Limited access to paid virtual applications - It is diﬃcult to provide a personalized nutritional advice using social media due to lack of comprehensive nutritional and physical measurements - No barriers - I do not use social media in my nutrition related practice - Other (please specify)…………… |
| **33. In your opinion, what is/are the beneﬁt/s of using social media/mass media/platforms in your nutrition-related practice? Select all that apply.**   - Quick and easy exchange of information - Networking with peer professionals (e.g. webinars, online lectures, online conferences) - It can reach large number of people in a short period of time - Cost-eﬀective - Time and location ﬂexibility - I do not use social media/mass media/platforms in my nutrition-related practice - Other (please specify)………. |  |
